# Antineutrophil Cytoplasmic Antibody–Negative Pauci-Immune Glomerulonephritis: Still Many Unknowns

**DOI:** 10.1016/j.ekir.2025.05.031

**Published:** 2025-05-23

**Authors:** Balazs Odler, David R.W. Jayne, Ingeborg M. Bajema

**Affiliations:** 1 Division of Nephrology, Department of Internal Medicine, Medical University of Graz, Graz, Austria; 2 Department of Medicine, University of Cambridge, Cambridge, UK; 3 Department of Pathology and Medical Biology, University Medical Center Groningen, Groningen, The Netherlands

See Clinical Research on Page 1450

Antineutrophil cytoplasmic antibody (ANCA)-negative vasculitis must be the most challenging entity in the vasculitis field, shedding doubt on whether ANCA are truly pathogenic (*O yes they are!*),^1^ because ANCA-negative patients provide proof for the exact same clinical and histological features being present in the absence of ANCA; at least, as determined by conventional testing. The latter consideration of course raises the question of whether in some patients, ANCA are present, but we are just not able to detect them, which in its turn, could mean that in some patients, ANCA are of certain subtypes that regular testing does not capture, for instance, ANCA directed against elastase. It is without doubt that ANCA-negative pauci-immune glomerulonephritis (GN) presents unique challenges in diagnosis and treatment.

In a recent issue of KI Reports, Floyd *et al.*^2^ report findings of one of the largest group of ANCA-negative patients thus far described, elegantly comparing them with a control group of ANCA-positive patients diagnosed closely to the patient group of interest. In comparison with ANCA-positive patients, ANCA-negative patients are younger, present more frequently with renal-limited diseases, and have worse estimated glomerular filtration rate at diagnosis and higher proteinuria. Some of these findings raise questions with regard to the time of diagnosis: are ANCA-negative patients diagnosed later because of the lack of extrarenal symptoms that in ANCA-positive patients may serve as alarm symptoms, which in turn accounts for the worse estimated glomerular filtration rate and higher proteinuria at diagnosis?

The most compelling question that lingers about the work is the one about definition and in relation to that, the long-lasting debate whether ANCA-negative vasculitis really exists. Because of its rare occurrence, Floyd *et al.*^2^ had to gather patients from 15 different international centers. It would be of interest to make an inventory of how the diagnosis was recorded in the original clinical work-up of the patients and what other diagnoses had been considered, details of the ANCA tests performed, which assays, and whether they were repeated. We know that “false negative” ANCA testing occurs. For now, the entry criterium for patients in this study heavily relies on the kidney biopsy findings defined by a pauci-immune GN; and this also harbors its most important flaw. Floyd *et al.*^2^ had to rely on the original biopsy reports because this study does not have a central pathology review. Therefore, what are the differential diagnoses based on histology? A crescentic necrotizing pattern of injury can occur in many different diseases, and it is mainly the immunofluorescence or immunohistochemistry findings that drive the pathology toward the histological diagnosis. Although the pauci-immune pattern gives the impressions of a well-defined entity, a “paucity” of deposits also occurs in other crescentic glomerulonephritides such as C3 GN; postinfectious GN; and a variety of small print entities, some of which are only be identified by electron microscopy. In the cohort described by Floyd *et al.*,^2^ it is mentioned that electron microscopy was performed, but not in how many patients. For immunofluorescence or immunohistochemistry, the study had to rely on descriptions from the original report. It would be of great additional value to perform a follow-up study with a central review of the biopsies to dig deeper into the subtleties of what typically defines pauci-immune GN in ANCA-negative patients and to make sure that even rarer entities were not missed.

Floyd *et al.*^2^ elegantly demonstrate that the treatment of patients with pauci-immune GN remains challenging. Although neutrophils play a central role in the pathogenesis of this entity, the exact mechanism beyond their activation is unclear. The absence of a clear serologic target limits the use of therapies such as rituximab, which are designed to deplete B cells producing ANCA, or plasma exchange. The long-term use of cyclophosphamide and glucocorticoids is associated with an increased risk of adverse events and toxicity, and their use is therefore limited. ANCA-associated vasculitis (AAV) often requires long-term immunosuppressive treatment to maintain remission, while the return of B cells, rising ANCA, and persistent T-cell dysregulation during remission are associated with disease relapse.^3^^,^^4^ In line, this relapse risk may be driven by a complex, intimate relationship between T and B cells. In ANCA-negative pauci-immune GN, there is lack of data on this crosstalk between T and B cells, which may explain, at least in part, the observation of lower relapse rates and less need of remission maintenance treatment. This is consistent with persisting ANCA being associated with higher relapse risk. Although, alternative antibodies that activate neutrophils cannot be excluded from the pathogenesis of pauci-immune GN,^5^ the involvement of other pathways seems also logical.

The present study did not include ANCA-negative patients receiving complement-targeted therapies; however, understanding the mechanisms of complement activation in this context may lead to more targeted and effective therapeutic strategies, especially in the context of similarities in clinical characteristics to myeloperoxidase-specific AAV. Unlike classical complement activation, which is typically antibody-dependent, the alternative pathway can be activated in the absence of specific antibodies, leading to a cascade of inflammatory events.^6^ In ANCA-negative pauci-immune GN, proteomic analyses revealed more pronounced alternative pathway activation,^7^ whereas low levels of serum C3 was associated with higher histopathological activity and worse treatment outcomes.^8^ This activation may lead to the generation of C5a, a potent neutrophil chemoattractant that can exacerbate neutrophil-mediated tissue injury. In myeloperoxidase-specific AAV, reliable evidence, mainly from murine models, suggests that the alternative pathway may be more involved in its pathogenesis, acting as an amplifier of ANCA-induced neutrophil activation and vascular injury.^9^^,^S1 In contrast, proteinase 3-specific AAV is more likely to be characterized by granulomatous and T-cell–rich inflammation with less robust evidence on the involvement of the complement pathway.S1 In addition, a greater sensitivity of patients with myeloperoxidase-specific AAV to avacopan, a C5aR agonist, was observed in the ADVOCATE trial.S2 Because targeting the complement system is already being successfully used in the treatment of AAV, its application may be beneficial in pauci-immune GN and the early use of such a treatment strategy may result in better control of inflammation in renal tissue at an early stage. This is essential because these patients have worse kidney outcomes compared to those with AAV, as we see in the current analysis by Floyd *et al.*^2^

Comorbidities, especially cardiovascular involvement and its risk factors, are an important issue when discussing the management of AAV.S3 The present study showed less extensive extrarenal manifestations in ANCA-negative patients; however, no data on comorbidities were reported and thus, whether these patients are at higher risk remains unclear. Similarly, data on disease- and treatment-related adverse events, such as serious infections or thrombotic events need more attention in the future.

In conclusion, this study raises the question of whether ANCA-negative pauci-immune GN may represent a distinct disease entity that may require unique approach to treatment and disease management. There is a clear need to include these patients in future studies, including clinical trials, although further data on thorough characterization of histopathological and clinical features are essential to improve their clinical outcomes. Nevertheless, the use of novel treatment options, such as complement-targeted therapies might be beneficial. The application of in-depth molecular analysis, leveraging cutting-edge molecular methods and artificial intelligence solutions, offers powerful tools to solve these uncertainties; however, international collaborations are essential given the rarity of this form of GN.

## Disclosure

BO reports receiving fees from AstraZeneca, CSL Vifor, Delta4, Glaxo Smith Kline, Novartis, and Otsuka and research grants from CSL Vifor and Otsuka, outside of the submitted work. DRWJ reports receiving fees from Aurinia, Alentis, Astra Zeneca, Chinook, CSL Vifor, Fate, Glaxo Smith Kline, Kissei, Novartis and Takeda. IMB reports receiving consulting fees from Alentis and CSL Vifor.

## Supplementary Material

Supplementary File (PDF) **Supplementary References**.

## Footnotes

Supplementary File (PDF)

**Supplementary References.**
