# Supplementary material for: Antineutrophil Cytoplasmic Antibody–Negative Pauci-Immune Glomerulonephritis: Still Many Unknowns
Source: Kidney Int Rep. 2025 May 23;10(7):2113–5. doi: 10.1016/j.ekir.2025.05.031 (PMC12266270; doi:10.1016/j.ekir.2025.05.031)
Supplement: Supplementary File (PDF) — Supplementary References. [file mmc1.pdf]

Supplemental references

S1. Chen M, Jayne DRW, Zhao MH. Complement in ANCA-associated vasculitis: mechanisms and implications for management. *Nat Rev Nephrol*. 2017;13(6):359-367. doi:10.1038/nrneph.2017.37

S2. Jayne DRW, Merkel PA, Schall TJ, Bekker P, ADVOCATE Study Group. Avacopan for the Treatment of ANCA-Associated Vasculitis. *N Engl J Med*. 2021;384(7):599-609. doi:10.1056/NEJMoa2023386

S3. Kronbichler A, Leierer J, Gauckler P, Shin JI. Comorbidities in ANCA-associated vasculitis. *Rheumatol Oxf Engl*. 2020;59(Suppl 3):iii79-iii83. doi:10.1093/rheumatology/kez617
